# Intrastromal injection of voriconazole as a therapeutic of fungal hypopyon: a case report

**DOI:** 10.11604/pamj.2020.35.52.20225

**Published:** 2020-02-21

**Authors:** Nabil Albab, Mohammed Oujidi, Sarah Belghamaidi, Jihane Hakam, Ibtissam Hajji, Redouane Moutaj, Abdeljalil Moutaouakil

**Affiliations:** 1 Department of Ophthalmology, Mohamed VI University Hospital of Marrakech, Marrakech, Morocco; 2 Department of Parasitology, Military Hospital Avicenne, Mohamed VI University Hospital of Marrakech, Marrakech, Morocco

**Keywords:** Intrastromal injection, voriconazole, fungal hypopyon

## Abstract

We describe the case of a 7-year-old child, victim of ocular trauma causing a corneal wound that was sutured urgently. The child has been regularly monitored in our department. Few months later, he consults for a painful red eye evolving within two weeks. The use of voriconazole as an intracameral injection has shown its superiority in treating fungal endophtalmitis. Further studies should be underwent to learn more about better injection strategies and so as to consider better its benefits and side effects.

## Introduction

Fungal infections of the cornea or of the anterior chamber are a rare case, challenging to manage, which causes several complications specifically the blindness. In our study we describe a case of a 7 year old child, for whom we used intracameral and intrastromal injections of voriconazole.

## Patient and observation

**Figure 1:**
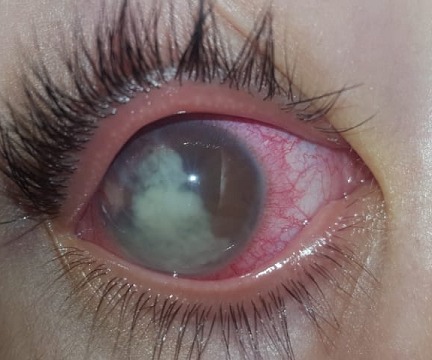
Image showing the presence of a total hypopyon in the anterior chamber

**Figure 2:**
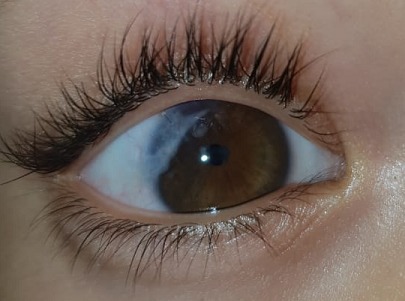
Image of hypopion regression after 2 weeks of treatment

We describe the case of a 7-year-old child, victim of ocular trauma causing a corneal wound that was sutured urgently. The child has been regularly monitored in our department. Few months later, he consults for a painful red eye evolving within two weeks. Slit lamp examination revealed a visual acuity limited to hand movements and conjunctival hyperemia. The cornea was clear, however we objected the presence of a total hypopyon in the anterior chamber (Figure 1). The lens and the fundus were inaccessible to examination. We performed a puncture of the anterior chamber into the operating room. The puncture liquid was sent to the laboratory. After that, we injected 0,05ml of voriconazole (VFEND, Pfizer Inc, New York, USA) concentrated to 50 micrograms/0.1 ml intracamerally. The puncture fluid being analyzed in the laboratory, revealed the presence of amoebiasis. Post intrastromal injection, the patient underwent a local treatment with topical antifungal therapy. He was examinated every two days and the response to the therapy was recorded. His best corrected visual acuity (BCVA) went from hand movements to 2/10. The hypopyon regressed in two weeks until complete disappearance (Figure 2). The infection was considered resolved when there was complete healing of the hypopyon and of the anterior chamber inflammation. The patient continued using topical antifungal therapy for next two weeks after complete healing.

## Discussion

Fungal infections of the cornea or of the anterior chamber are major cause of blindness in the world [1]. They are usually challenging to manage [2]. In the last few years, broad spectrum antifungal agents such as voriconazole have been tried and also alternate routes of administration such as intracorneal injections have been used to treat deep and/or resistant fungal keratitis [3]. However, the penetration of many antifungal drugs into the cornea being suboptimal, it makes it difficult to treat cases of deep mycotic keratitis. To overcome these problems, investigators have evaluated alternate routes such as the use of intracameral and intrastromal injections of voriconazole [4]. Voriconazole has the best in vitro efficacy against pathogenic fungi compared with other agents and it has low minimum inhibitory concentration (MIC) values against Candida [5]. In our case, Voriconazole 50 microgramm/0.1 mL was injected in the anterior chamber after ponction of the hypopyon. We believe that this technique of injection, has the best chance of achieving MIC of the drug at the tissue level. Only few preliminary clinical studies have reported the effect of intracameral injection of Voriconazole on deep keratomycosis and endophtalmitis and seems to be safe and effective [6]. The experimental use of intracameral voriconazole in humans showed no toxic effects when the aqueous concentration was 10 μg/mL - 1.5 mg/mL, above 1.5 mg/mL there was a dose-dependant reduction in corneal endothelial cells, trabecular meshwork cells, and retinal pigment epithelial cells [7]. This medical therapy was safe, allowing higher drug bioavailability within the anterior chamber thus controlling the intraocular infection compared to topical use. However, clinical trials assessing the safety and efficacy of intracameral voriconazole should be considered [8].

## Conclusion

The use of voriconazole as an intracameral injection has shown its superiority in treating fungal endophtalmitis. Further studies should be underwent to learn more about better injection strategies and so as to consider better its benefits and side effects.

## Competing interests

The authors declare no competing interests.
